# Almond Anthracnose: Current Knowledge and Future Perspectives

**DOI:** 10.3390/plants9080945

**Published:** 2020-07-27

**Authors:** Ana López-Moral, Carlos Agustí-Brisach, María Lovera, Octavio Arquero, Antonio Trapero

**Affiliations:** 1 Departamento de Agronomía, ETSIAM, Universidad de Córdoba, Campus de Rabanales, Edif. C4, 14071 Córdoba, Spain; b92lomoa@uco.es (A.L.-M.); cagusti@uco.es (C.A.-B.); 2 Departamento de Fruticultura Mediterránea, IFAPA, Alameda del obispo, 14004 Córdoba, Spain; maria.lovera@juntadeandalucia.es (M.L.); octavio.arquero@juntadeandalucia.es (O.A.)

**Keywords:** etiology, causal agents, *Colletotrichum* spp., epidemiology, *Prunus dulcis*, management strategies

## Abstract

Almond anthracnose caused by *Colletotrichum* spp. has been described as one of the most important diseases of this nut crop in the main almond-growing regions worldwide, including California, Australia and Spain. Currently, almond anthracnose is considered a re-emerging disease in the countries across the Mediterranean Basin due to the shift of plantations from the original crop areas to others with climatic, edaphic and orographic conditions favoring crop growing and yield. The pathogen mainly affects fruit at the youngest maturity stages, causing depressed, round and orange or brown lesions with abundant gum. The affected fruits can fall prematurely and lead to the drying of branches, causing significant economic losses in years of epidemics. This review aims to compile the current knowledge on the etiology, epidemiology and management of this disease.

## 1. Introduction

Almond [*Prunus dulcis* (Mill.) D.A. Webb] belongs to Rosaceae. It is the only species of the *Prunus* genus whose commercial interest lies in its seeds, whereas the remaining *Prunus* species are mainly grown for their juicy flesh or mesocarp (stone fruits). For this reason, almond is classified as a nut rather than a stone fruit despite having major genetic similarities with the remaining *Prunus* species [1]. It represents one of the first tree nuts domesticated by humans, probably because almond kernels represent a nutritious, compact and relatively non-perishable food source. To date, the almond crop is widely distributed across the five continents of the world, from Asia to the Mediterranean Basin, Australia, North and South America and South Africa [2].

In particular, almond is a traditional and characteristic crop in the Mediterranean Basin, with great social and economic importance due to its large acreage and its demarcation, mostly in areas with unfavorable climatic and orographic conditions [3]. Based on surface area, Spain currently leads the world area in almond production, with more than 650,000 ha, followed by the USA with over 440,000 ha in 2018 [3,4]. However, the USA leads the world in almond production with nearly two million tons of shelled almonds and an average yield of approximately 2500 kg of almond kernel per ha, while the almond production in Spain is nearly 340,000 tons of shelled almonds with an average yield of approximately 150 kg of almond kernels per ha [4].

As we mentioned above, almond has been traditionally associated with marginal growing areas across the Mediterranean Basin characterized by unfavorable climatic, edaphic and orographic conditions [3]. However, almond has been introduced as a potential alternative crop in non-traditional almond-growing regions, in areas characterized by moderate temperatures in spring–summer and high relative humidity (RH). Thus, a shift from traditional dry-farming cultivation systems to more intensive almond cropping systems based on high-input strategies has been observed progressively. For example, we find these two scenarios across the southern Iberian Peninsula: (i) traditional, marginal almond growing areas (provinces of Almeria and Granada) and (ii) novel, intensive almond growing areas with climatic, edaphic and orographic conditions favoring crop growing and yield across the Guadalquivir valley (provinces of Huelva and Sevilla, mainly). Therefore, the extension of the almond crop from the first scenario to the second one has been conducted with the objective of enhancing the competitiveness of almond production in the global market, i.e., markedly increasing almond kernel production [3,5,6].

However, this forced change in almond crop management and the environmental conditions of the new almond-growing regions favor an increase in the incidence and prevalence of foliar and fruit diseases, limiting its profitability. Among the aerial diseases that affect this crop, the most common are leaf blotch (*Polystigma amygdalinum* P.F. Cannon), shot hole [*Wilsonomyces carpophilus* (Lév.) Adask., J.M. Ogawa & E.E. Butler], blossom blight (*Monilia* spp.), leaf curl [*Taphrina deformans* (Berk.) Tul.] and anthracnose (*Colletotrichum* spp.) [5,6,7]. The last one is considered a major disease of this crop worldwide causing important economic losses when serious outbreaks occur [8]. Nevertheless, almond anthracnose (AA) has been considered a secondary disease in specific almond-growing areas across the Mediterranean Basin (i.e., Italy and southern Spain) until its recently re-emergence as a consequence of the new establishment of almond plantings in non-traditional almond-growing areas [9,10].

Due to the importance of AA in all the almond growing areas of the world as well as its re-emergence in several Mediterranean countries, this review aims to provide a current synthesis of the history, distribution, symptomatology, etiology, epidemiology and management of the disease.

## 2. History and Distribution

Symptoms of AA were observed for the first time in Sardinia (Italy) in 1896 [11] and in 1900 in Mallorca (Spain) [12]. Subsequently, in 1915, Doidge recorded the occurrence of the disease on green almonds for the first time in South Africa, in almond orchards located in the Paarl district [13]. The field observations and fungal isolations from affected almonds conducted across this country suggested that the disease was extended to most of the almond growing areas in South Africa including the Caledon, Stellenbosch, Paarl, Wellington, Tulbagh, Malmesbury and Piquetberg and districts. Fifteen years later, a major outbreak of the disease occurred in the Western Cape province (South Africa), where almond growers suffered important economic losses [14]. It was then that the disease was commonly called “gumming of the almonds”, “gummosis” or “kernel rot” due to the characteristic symptoms presented by the affected fruit [12,14]. In parallel with the first report of the disease in South Africa, it was described in 1916 as a likely new disease of almond in America [15]. Since then, major damage was apparently not been recorded from any other part of the world until 1978, when AA was described first in Israel as causing important economic losses that were prolonged through the 1980s [8,16]. In California, it was rare until the late 1980s, causing heavy losses in the 1990s [8,17]. In Australia, AA was first reported in 1998 [18]. The disease has also been described in the remaining almond growing areas worldwide including France, Greece, Italy, Morocco, Tunisia and Spain [8,17,19]. It is interesting to note that AA was considered a secondary disease in the Mediterranean Basin until the 1960s. This was already referenced in 1900 on the island of Mallorca (Spain) [12], but the causal agent of the disease was not identified in this country until 1965 in Huesca (northeastern Spain). Since then, the disease has been detected regularly in commercial orchards across the Guadalquivir valley, showing higher outbreaks with favorable environmental conditions for disease development. The latest major outbreak of AA was reported in spring of 2014 in southern Spain (provinces of Huelva and Sevilla), when the disease was considered as re-emerging in this country [9].

## 3. Symptomatology

**Figure 1:**
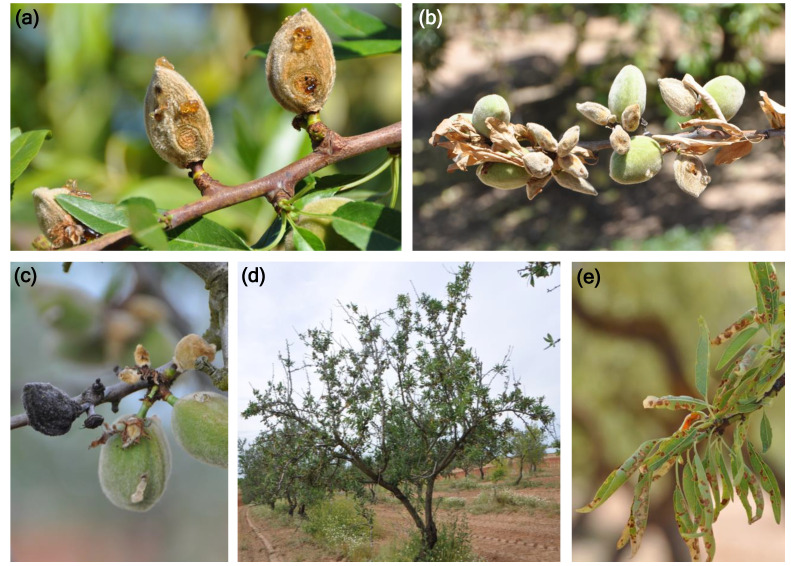
Characteristic symptoms of almond anthracnose caused by *Colletotrichum* spp. (**a**) depressed, sunken, round and orange lesions on green almonds; (**b**) branch with mummified fruits and necrotic leaves; (**c**) mummified fruits from infections caused the previous year and remaining in the tree canopy; (**d**) defoliation and dieback of shoots and branches as a consequence of the toxins produced by the pathogen; (**e**) necrotic irregular lesions in the tips and margins of the leaves [9].

The pathogen affects mainly almond fruit, but flowers, leaves and woody tissues can also be affected. Infected flowers become blighted, often with orange droplets of conidia on the floral cup [17]. However, the most characteristic symptoms of the disease are observed in green fruits. The infected fruit show depressed, round and orange or brown lesions from 5 to 12 mm in diameter that develop on the fruit surface in spring–summer and produce abundant gum (Figure 1a). The diagnosis of the disease is difficult in the incipient lesions since the color of affected areas does not clearly differ from the asymptomatic epidermis. Whenever symptoms progress, abundant whitish mycelium and orange masses of conidia are produced on the surface of infected fruit. Subsequently, fruit mummify (Figure 1b) and fall prematurely to the soil [9,17,20,21]. The mummies that remain in the tree canopy during autumn and winter will be the main inoculum source for infection in the following year (Figure 1c). Although the pathogen causes mainly fruit rot, a secondary syndrome consisting of leaf necrosis, defoliation, shoot blight and branch dieback has also been observed in seriously affected trees (Figure 1d) [9,20,22]. Leaves show necrosis starting from the tips and margins and extending to the entire leaf blade (Figure 1e) [23]. This secondary syndrome seems to be caused by the translocation of the toxins produced by the pathogen in the affected fruit remaining in the tree canopy. The pathogen can be isolated consistently from the affected tissues of almond fruit. However, it cannot be isolated from necrotic leaves as well as from the wood of shoots and branches showing blight and dieback, probably because this syndrome is caused directly by the phytotoxins, but the pathogen is no longer present in the tissues [9,20,22].

## 4. Causal Agent

The causal agent of the disease was described for the first time in Italy in 1896 as *Gloeosporium amygdalinum* Brizi [11]. Around twenty years later, *G. amygdalinum* was also reported as the causal agent of the disease in South Africa [13] and America [15]. Due to subsequent studies that confirmed that species belonging to *Gloeosporium* genus were morphologically indistinguishable, *G. amygdalinum* was reclassified as *Colletotrichum gloeosporioides* (Penz.) Penz. & Sacc [24]. In 1965, a new *Colletotrichum* species close to—but distinct from—*C. gloeosporioides*, was identified in Australia as *C. acutatum* J.H. Simmonds mainly on the basis of morphologic differences in the ends of conidia, which were sharp in *C. acutatum* and rounded in *C. gloeosporioides* [25,26,27]. Consequently, based on these morphologic characters, most of the isolates of *C. gloeosporioides* species complex associated with AA worldwide were reclassified as *C. acutatum* [9], with the exception of only the isolates from Israel that had unique morphologic characteristics remained as *C. gloeosporioides* [28].

Subsequently, molecular analyses of *Colletotrichum* isolates from almond were useful to confirm the identity of the isolates from Australia and California as *C. acutatum* [29,30,31], while the isolates from Israel were reclassified as *C. acutatum* [16]. However, *C. acutatum* has recently been considered as a species complex since it is an extremely variable genetic species. To date, 34 phylogenetic species have been accepted within the *C. acutatum* species complex [*C. acutatum sensu lato* (*s.l.*)] [32,33]. Therefore, all the *Colletotrichum* isolates associated with AA over the world were identified as phylogenetic species within *C. acutatum* species complex [*C. acutatum sensu lato* (*s.l.*)] including those from Australia, California, Israel and Spain. Among them, *C. fioriniae* Marcelino & Gouli ex R.G. Shivas & Y.P. Tan (pink colony subpopulation) and *C. godetiae* Neerg. (syn. *C. clavatum* Agosteo, Fedda & Cacciola; gray colony subpopulation [34]) have been considered the prevalent species causing AA in the main almond-growing regions of the world such as California [8,30] and Australia [18,31]. Additionally, *C. acutatum sensu stricto* (*s.s*.) has also been reported as a causal agent of AA in South Australia [35].

More recently, López-Moral et al. [9] characterized the *Colletotrichum* isolates associated with AA in southern Spain since a serious outbreak of the disease was observed for the first time in spring of 2014 in commercial almond orchards in the provinces of Huelva and Sevilla (Andalusia region). In this study, *C. acutatum* s.s. and *C. godetiae* were reported as causal agents of AA in Spain, with *C. godetiae* being the most common species. The frequency of *C. godetiae* isolated from affected almonds was markedly higher (89.3%) than that of *C. acutatum s.s.* (10.7%) [9]. *Colletotrichum godetiae* is also the prevalent *Colletotrichum* species associated with olive anthracnose in the Andalusia region [36].

It is interesting to note that the morphologic and molecular characterization of the Andalusian *Colletotrichum* isolates from almond revealed that the species *C. acutatum s.s.* described by López-Moral et al. [9], which showed pink–orange colonies, did not coincide with the pink subpopulations described in other countries, which were identified as *C. fioriniae* [30,32]. On the other hand, the colonies identified as *C. godetiae* from almond in Spain coincided with the gray subpopulation associated with AA worldwide [9,30,32].

**Figure 2:**
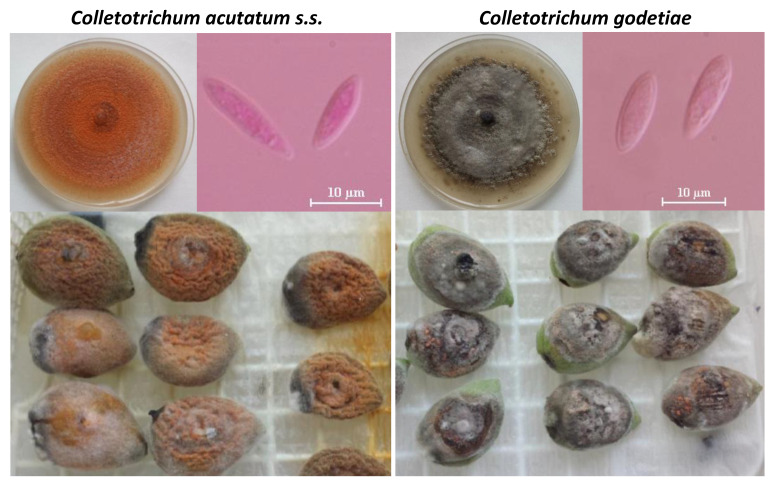
Colonies and conidia of *Colletotrichum acutatum s.s.* (isolate Col-506; pink–orange subpopulation) and *C. godetiae* (isolate Col-522; gray subpopulation) identified as causal agents of almond anthracnose in Southern Spain. Colonies grown on PDA and on inoculated almond fruits at 23 ± 2 °C with a 12-h photoperiod for 7 and 14 days, respectively. Scale bars: (conidia) 10 μm.

In general, fungal colonies of *Colletotrichum* spp. associated with AA show radial growth with concentric circles and abundant aerial mycelium on potato dextrose agar (PDA) when they are incubated at 23 ± 2 °C with a 12-h photoperiod. Colony color varies from light to dark gray for *C. godetiae* isolates (gray subpopulation); and from salmon to pink–orange for *C. acutatum s.s. (*Figure 2*).* or *C. fioriniae* isolates (pink–orange subpopulations) [9,20,28]. The mycelial growth rate ranges from 3.0 to 6.0 mm/day on PDA at 25 °C [9,20]. Interestingly, López-Moral et al. [9] observed that the colony colors developed on almond fruits artificially inoculated by *C. godetiae* and *C. acutatum s.s*. isolates from almond in Spain coincided with the colors of their colonies grown on PDA, being gray or pink–orange for *C. godetiae* and *C. acutatum s.s.* isolates, respectively (Figure 2). In spite of conidium morphology may vary with the *Colletotrichum* species, the morphology of conidia of *Colletotrichum* isolates from almond is not useful to distinguish them between species. In general, all *Colletotrichum* species associated with AA show unicellular, hyaline conidia with two sharp ends. Interestingly, conidia of *C. godetiae* from almond show similar morphology to those of *C. acutatum* from almond (two sharp ends in both cases), whereas conidia of *C. godetiae* from other hosts such as olive usually show a single sharp end [9] (Figure 2). However, this morphologic differences on conidium morphology between isolates of *C. godetiae* from different hosts do not result in genetic differences when using six genomic regions [9].

## 5. Disease Cycle and Epidemiology

AA has been described as a polycyclic disease. The pathogen overwinters mainly in mummified fruits remaining in the tree canopy. The inoculum surviving in mummified fruits will be the primary inoculum for the infection of the next crop season. The primary infections begin during late winter to early spring, when the first raining events occur at the beginning of the year. Fungal sporulation, infection and disease development are favored by wet weather and mild temperatures from 10 to 25 °C. Conidia are dispersed by splashing rain, and infections could occur through fruit, leaf or petal tissues, with the youngest fruit being the most vulnerable tissues to the infection. The pathogen develops orange gelatinous masses on the infected fruits with abundant conidia, which serve as inoculum for the continuous secondary infections that occur during spring until the rains cease. The infected fruit mummify and most of them fall prematurely to the soil [8,9,21].

In the past two decades, studies on the epidemiology of AA have been conducted worldwide in order to elucidate the main biotic (i.e., *Colletotrichum* subpopulations, age of almond tissues, etc.) and abiotic (i.e., pH, temperature, wetness, etc.) factors involved in the disease cycle [10,20,28,37,38,39,40,41,42,43]. A compilation of all these studies with regards to their effect on the different parts of the disease cycle are described below.

### 5.1. Dispersal and Survival

The pathogen produces conidia from acervuli in the infected tissues, which are spread by splash-raining. The abundant conidial production suggests that the fungus uses the nutrients on the plant tissues instead of the reserves from the original conidium for reproducing [21]. Although this fact could indicate that the fungus is growing as an epiphyte, the capacity of the pathogen to overwinter in the mummified almond fruit that remain in the tree canopy suggest that quiescent infections are needed for pathogen survival. McKay et al. [43] recovered *Colletotrichum* from mummified fruit, peduncles and bark from almond monthly throughout a year-long sampling period suggesting all these tissues as potential inoculum sources of the pathogen in Australia. These same authors also recovered *Colletotrichum* from asymptomatic leaves, fruit, bark, buds and blossom, but with less consistency of isolation and frequency than from mummified fruits and peduncles [43].

*Colletotrichum* species could also survive in alternative hosts. Previous studies conducted in horticultural hosts demonstrated that weeds (i.e., *Coniza* sp.; *Vicia* sp.) could serve as potential alternative host of the pathogen, since it may live as an endophyte of these hosts spreading to adjacent susceptible plants [21]. Even though to date there are no references for the role of weeds as potential alternative hosts of *Colletotrichum* spp. associated with AA, special attention must be given to this fact for future research.

### 5.2. Infection and Disease Progress

Infections could occur through fruit, leaf or petal tissues, with fruit likely being the main route of almond infection. The vulnerability of fruit to *Colletotrichum* infections is higher in the first stage of fruit development, in particular at the fruitlet stage [10,14,28,39,40]. Recently, López-Moral et al. [10] evaluated the effect of fruit maturity on *Colletotrichum* infections using detached almond fruit from four different maturity stages between the fruitlet stage and the end of the maturity stage. These authors demonstrated that the susceptibility of the fruit decreased significantly with increasing maturity, with the fruitlet stage being the most susceptible.

In previous studies, Freeman et al. [28] emphasized that *Colletotrichum* spp. associated with AA infected only at initial fruiting stages, but do not attack other plant organs. In contrast, studies conducted in California by Diéguez-Uribeondo et al. [41] demonstrated that conidia of *Colletotrichum* spp. could also infect leaves and flowers. In fact, these authors demonstrated that the conidia of the fungus germinate more rapidly on petals than on almond leaves, also showing longer germ tubes under laboratory-controlled conditions. However, almond infections by *Colletotrichum* through blossoms in field conditions have not been demonstrated yet. On the other hand, the effect of leaf age on *Colletotrichum* infections has also been recently evaluated by López-Moral et al. [10] using detached leaves collected at approximately 1 and 3 months after bud expansion, with the youngest ones being the most susceptible to the infection.

To sum up, the observations conducted by Diéguez-Uribeondo et al. [41] on both petal and leaf tissues, suggest that the fungus penetrates by means of appressoria, and the host colonization is first subcuticular and then intracellular. Therefore, the colonization of almond tissues by *Colletotrichum* is described as subcuticular–intracellular hemibiotrophy and intercellular necrotrophy.

With respect to the infection based on field observations, Shabi and Katan [19] monitored the disease progression under field conditions along six consecutive seasons, showing that there was a close relationship between infection and spring rains. Disease incidence was lower in seasons with dry springs compared to others with wet springs.

### 5.3. Host-Pathogen Interactions

Micro-environmental conditions within the host plant can play an important role in the infection and development of the pathogen, and the subsequent plant–pathogen interactions [42]. In this context, several studies have been conducted to determine the effect of temperature, wetness duration, kernel moisture content or pH on *Colletotrichum* infections in almond [9,20,44].

All in vitro tests conducted to evaluate the effect of temperature on mycelial growth of *Colletotrichum* isolates associated with AA showed that they are able to grow at temperatures ranging from 10 to 30 °C, showing optimum growth at approximately 25 °C [9,20]. It is interesting to note that fungal isolates from the gray and pink–orange subpopulations, or from different hosts were used in these studies for comparative purposes. The studies conducted with the Californian subpopulations of *Colletotrichum* isolates did not find significant differences in growth rate between them at 20–25 °C, while the gray cultures (*C. godetiae*) grew faster at 10 and 15 °C than pink–orange subpopulations (*C. acutatum s.l.*). However, growth of gray cultures stopped at 32 °C, whereas pink cultures still grew at this temperature [20]. The studies conducted with the Spanish subpopulations of *Colletotrichum* isolates, showed that the gray cultures (*C. godetiae*) from almond have an optimum growth temperature that is significantly lower than the rest of the isolates tested [pink–orange subpopulation from almond (*C. acutatum s.s*.) and *C. godetiae* and *C. nymphaeae* from olive)]. Finally, *C. godetiae* from almond also grew at temperatures below 5 °C, while the rest of the isolates were not able to grow below 4–5 °C [9]. Diéguez-Uribeondo et al. [44] evaluated the effect of wetness duration and temperature on the infection of blossoms, leaves, fruit and woody tissues of almond by *C. acutatum* under laboratory conditions. This study revealed that longer wetness durations are needed for the infection of leaves than for blossoms, with the disease increasing linearly with increasing wetness duration. On the other hand, the temperature of incubation affected the rate of disease development, while final disease levels were very similar at 10, 15 or 20 °C. Moreover, these same authors also evaluated the effect of temperature on the infection of four different almond cultivars (‘Carmel’, ‘Ne Plus Ultra’, ‘Nonpareil’ and ‘Wood Colony’) showing that the cultivar resistance varied depending on the inoculated tissue and on the temperature of incubation [44]. More recently, the effect of temperature on the infection of *Colletotrichum* subpopulations has also been evaluated on detached almond fruit by López-Moral et al. [10]. In this recent study, the infection and progress of both gray (*C. godetiae*) and pink–orange (*C. acutatum s.s*.) subpopulations was observed on inoculated fruit incubated at all temperatures (from 10 to 30 °C), with disease development being higher at 25 °C. Significant differences in virulence were found between *Colletotrichum* subpopulations, with *C. acutatum s.s*. isolates being more virulent than *C. godetiae* isolates [10]. These results suggest a greater adaptability of *Colletotrichum* subpopulations from almond at different temperatures.

On the other hand, plant pathogenic fungi can modulate the host pH environment creating their own conducive microenvironment that favors the establishment of infections [45]. With respect to almond infections, Diéguez-Uribeondo et al. [42] demonstrated that *Colletotrichum* isolates associated with AA are able to induce an alkaline modulation of almond tissues due to production of ammonia from very early to later stages of pathogen colonization. This pH modulation was observed in localized areas associated with fungal structures (i.e., appressoria, infection vesicles, intra- and intercellular hyphae) adjacent to and within host epidermal cells during colonization. Thus, these authors concluded that *Colletotrichum* spp. are able to produce ammonia in almond tissues after infection modulating the pH within almond epidermal tissues, leading to further colonization of the infected tissues [42].

Finally, the effect of kernel moisture content on growth of *Colletotrichum* isolates was evaluated to define environmental conditions that lead to postharvest kernel damage. For this purpose, kernels were placed in plastic containers with saturated solutions of KNO_3_, K_2_HPO_4_, KCl, NaCl and sucrose and inoculated with *Colletotrichum* isolates seven days later. The data obtained in this study revealed that the postharvest damage to almond kernel appears as a consequence of preharvest epidemics that were not managed in the field [20].

### 5.4. Host Specificity

Although it is common to find single *Colletotrichum* species infecting multiple hosts, the pathogenic specialization of *Colletotrichum* spp. on their hosts of origin has been a characteristic traditionally used in the identification of specific or intraspecific taxa in this genus [24,26]. Cross-infection potential has been reported among different species of *Colletotrichum* on a broad diversity of tropical, subtropical and temperate fruits under laboratory-controlled conditions [28]. In Israel, Freeman and Shabi [37] evaluated the cross-infection of a large number of fruit crops using isolates of *C. acutatum s.l*. (from apple, peach and pecan) and *C. gloeosporioides s.l*. (from almond, apple, avocado, mango and pecan). In this study, the *C. gloeosporioides* isolates from almond grew more slowly, causing significant smaller lesions on all inoculated fruit than the other isolates. In parallel, Freeman et al. [38] evaluated the host specificity of *C. gloeosporioides* from almond and avocado on fruits of both crops. The avocado isolates showed higher virulence on both avocado and almond fruits than the almond isolates. These differences in virulence observed between isolates from different hosts was due to the fact that they were all grouped first as *C. gloeosporioides* according to their morphology. Interestingly, a collection of *Colletotrichum* isolates from different hosts and origins including those used by Freeman et al. [38] were analyzed molecularly two years later by Freeman et al. [28]. It was demonstrated that some isolates previously grouped as *C. gloeosporioides* were identified as *C. acutatum s.l.* (i.e., isolate ALM-US-1A from almonds from California), whereas the identity of all the isolates from avocado was confirmed as *C. gloeosporioides* [28]. Freeman et al. [28] also evaluated cross-infections on detached fruits of avocado, almond, mango and nectarine using isolates of *C. acutatum s.l*. (from anemone, apple and peach) and *C. gloeosporioides s.l*. (from almond, apple, avocado and mango), demonstrating cross-infection between the two species of *Colletotrichum* and the fruit crops. More recently, López-Moral et al. [9] evaluated cross-infections on almond, apple and olive using isolates of *C. acutatum s.s*. from almond, *C. godetiae* from almond and olive and *C. nymphaeae* from olive, all from Spain. Their results showed differences in virulence and some degree of pathogenic specialization among isolates. In olives, the two *Colletotrichum* isolates from olive were more virulent than the *Colletotrichum* isolates from almond. In almond and apple, *C. acutatum s.s.* from almond did not differ in virulence from the olive isolates. Finally, in the three hosts tested, the *C. godetiae* isolates from almond were the least virulent. These authors concluded that the *Colletotrichum* spp. isolates from olive showed little or no pathogenic specialization, since they were able to infect and develop symptoms in other hosts with a similar degree of virulence to that in their host of origin [9]. Nevertheless, all of these studies were conducted under laboratory-controlled conditions, and further research should be conducted to demonstrate cross-infections under field conditions. In fact, Freeman et al. [28] tried to carry out isolation from naturally fruit infections, but their results provided no evidence that cross-infection occurs under natural field infections.

### 5.5. Intraspecific Relationships

It is well known that in almond there are two main *Colletotrichum* subpopulations associated with anthracnose worldwide: (i) the gray subpopulation [*C. gloeosporioides* (only in Israel); *(C) godetiae*]; and (ii) the pink–orange subpopulation [*C. fioriniae* or *C. acutatum s.s*. (only in Australia and Spain)]. Among them, the *Colletotrichum* species belonging to pink–orange subpopulations are significantly more virulent to almond fruit than those belonging to gray subpopulations [9,10,20,31]. However, to date, important differences in virulence on almond infections have not been described yet among isolates within species and among *Colletotrichum* species (or isolate) from different geographic origins based on morphologic, molecular and pathogenic characters with the exception of some previous studies [28,31]. Likewise, special attention should be given to this finding in future studies since anthracnose is associated with a broad range of species of *Colletotrichum* affecting many crops and differences in virulence between fungal species and between isolates within species from different hosts and geographic origins were described [32,36,46,47].

## 6. Management Strategies

The control of the disease must be based on an Integrated Pest Management (IPM) program preventing infections. Prevention is especially important in AA because *Colletotrichum* spp. are established slowly in the orchard over years, showing up when environmental conditions are favorable for disease outbreaks. Once disease symptoms appear, the control of AA would be difficult if the inoculum pressure in the field is high. Thus, preventive measures including cultural practices, cultivar resistance and biologic control must be considered to face *Colletotrichum* infections. Fungicide treatments should be used only to prevent economic losses in years of epidemics when preventive measures are not enough to control the disease or orchards with a disease history suggest that preventive treatments are required. All these management strategies as well as an update on the research studies concerning this topic are described in detail below.

### 6.1. Cultural Practices

Cultural practices are focused on the prevention of the disease mainly by reducing overwintering inoculum by removal of inoculum sources of the pathogen (i.e., mummified fruits, affected shoots and branches, etc.) and an appropriate management of irrigation, i.e., using low-angle sprinkler irrigation in order to reduce wetness in the tree canopy. Good pruning management helps to increase the aeration of the tree canopy and reduces the humidity on the plant tissues making difficult the infection and establishment of the pathogen. Additionally, adequate fertilization, control of other pests (i.e., insects, mites, etc.) and the eradication of potential alternative hosts growing in the field (i.e., weeds) also help maintain vigorous and healthy trees, promoting more resistance to the infection [17,22,48].

### 6.2. Cultivar Resistance

Cultivar resistance offers an economically and eco-friendly alternative to chemical control with minimal environmental impact in crop protection. Thus, the selection of resistant cultivars is essential to reducing the impact of the disease, and it is considered one of the most important management strategies in the context of sustainable agriculture. Tolerant cultivars (cvs.) are available for the common almond foliar diseases [6,10,17,22,49] and their selection depends on prevailing local disease problems among other agronomical, environmental and marketing considerations.

Several studies evaluating cultivar resistance against AA have been conducted in the past ninety years in the main almond-producing countries worldwide. To our knowledge, the first study on cultivar resistance of AA was conducted in South Africa in 1931 by Dippenaar [14]. This author evaluated the disease severity of AA for one epidemic season under natural field conditions on the almond cultivars grown in Western Cape. Dippenaar [14] observed that none of the cultivars were resistant to AA, with ‘Jordan’ and ‘Nonpareil’ being the most susceptible cultivars followed by ‘Paper-Shell’ and ‘I.X.L’. Studies conducted in Italy by Prota [50] suggested that ‘Grappolina’ was highly susceptible to AA. In France, Crossa-Raynaud [51] evaluated 21 almond cultivars that were classified in five categories depending on the percentage of the affected fruit surface. ‘Abiod’, ‘Cavaliera’, ‘Constantini’ and ‘NePlus Ultra’ were described as susceptible cultivars; ‘Marcona’, ‘Nonpareil’ and ‘Texas’ were a little susceptible; and ‘Fasciuneddu’ was resistant [51]. Some of these results were not in concordance with those obtained a few years later in Spain by Palazón and Palazón [23], who indicated that ‘Marcona’ was moderately susceptible to AA, showing similar levels of susceptibility to those observed in ‘Desmayo Largueta’. Striem et al. [39] evaluated the resistance of four almond cultivars to AA under field conditions in Israel. This study revealed that the selection ‘M.D.1’ was the most resistant one to the disease, followed by the selection ‘59/4’. In contrast, the almond cvs. Poria and Ne Plus Ultra resulted to be susceptible to AA [39].

Because much of this information on the susceptibility of almond varieties to anthracnose was outdated, and new varieties are continually being introduced from breeding programs [52,53,54,55,56,57,58], major studies on almond cultivar resistance against AA have been conducted during these last two decades [10,17,22]. These studies concluded that all commercial almond cultivars appear to be susceptible to anthracnose, but there are important differences in susceptibility between cultivars [10,17,22]. A compilation of the results of all these studies is described in detail below and in Table 1.

In California, the susceptibility to AA of the most common American almond cultivars was evaluated on blossoms, leaves and fruit tissues under field and laboratory-controlled conditions [17,44,49]. These authors indicated that ‘Nonpareil’ was significantly more tolerant to AA than other important cultivars used in California such as ‘Carmel’, ‘Ne Plus Ultra’ and ‘Wood Colony’, under both laboratory and natural field conditions [44]. According to the almond pest management guidelines described by Adaskaveg et al. [22] for the control of AA in California, the almond cultivars Thompson, Merced, Price, Peerless, Winters, Monterey, Fritz and Butte have been described as susceptible; ‘Harvey’, ‘Carmel’, ‘Ne Plus Ultra’, ‘Padre’ and ‘Mission’ are moderately susceptible; and ‘Nonpareil’ is considered the least susceptible cultivar to AA. Finally, Palacio-Bielsa et al. [17] described the susceptibility of 15 almond cultivars to AA, with 12 of them being common to those described by Adaskaveg et al. [22]. However, differences in the grouping of cultivars by susceptibility category were found between these two references. Three more cultivars were tested by Palacio-Bielsa et al. [17], including ‘Aldrich’ (moderately susceptible), ‘Drake’ (tolerant) and ‘Sonora’ (susceptible).

In South Australia, McKay et al. [43] monitored the progress of AA for three growing seasons in an almond orchard on the cvs. Price and Nonpareil and showed up to 80% of fruit affected by the disease in previous epidemic years, with ‘Nonpareil’ being more tolerant than ‘Price’ [43].

More recently, in Spain, López-Moral et al. [10] evaluated the resistance of 19 cultivars to AA under laboratory-controlled conditions on detached fruit and leaves. On the basis of inoculations on fruit, four categories of susceptibility (highly susceptible, susceptible, moderately susceptible and resistant or tolerant) were distinguished. Thus, ‘Ferraduel‘ and ‘Nonpareil‘ were classified as the most tolerant cultivars (resistant) and ‘Tarraco‘ and ‘Penta‘ as the most susceptible cultivars (highly susceptible). On leaf tissues, ‘Nonpareil’ was consistently the most tolerant cultivar [10]. These authors evaluated the correspondence of their laboratory results with the severity of the disease in the field for the seven common cultivars evaluated under both laboratory and field conditions. A significant positive linear correlation between field and laboratory observations was obtained, with ‘Ferraduel’ resulting in the most resistant cultivar and ‘Tarraco’ the most susceptible in field observations. Finally, these same authors noticed that ‘Guara’ and ‘Tuono’ showed identical values of disease severity on inoculated fruit [10]. Similarly, Ollero-Lara et al. [6] observed the same level of susceptibility of these two cvs. against four other almond foliar diseases, red leaf blotch, shot hole, blossom blight and leaf curl, in southern Spain. This similarity between these two cultivars in susceptibility to all the almond foliar diseases could be related to the results obtained by Dicenta et al. [59], who showed these two cultivars have identical genetic profiles.

### 6.3. Biologic Control

To date, there are no specific studies on biologic control against AA. All the studies concerning biologic control against *Colletotrichum* spp. have been conducted on anthracnose of other host plants such as apple, avocado, chili, mango, olive, papaya or strawberry [60,61,62,63,64,65].

In this context, bacterial strains of *Azospirillum brasilense* or *Bacillus subtilis* have been described as potential biologic controls agents (BCAs) against *C. acutatum* and *C. gloeosporioides* in strawberry [62] and mango or apple [61], respectively. In addition, Tortora et al. [62] observed that the endophytic strains of *A. brasilense* obtained from the roots of strawberry plants secrete catechol type siderophores, including salicylic acid, showing antifungal activity against *C. acutatum*.

On the other hand, the use of antagonistic yeasts as BCAs has been explored as a promising alternative to chemicals against fruit diseases [66]. In this context, screenings of broad collections of endophytic yeast strains have been conducted to evaluate their antagonistic effect against Colletotrichum diseases in different crops such as avocado [63], chili [60] and papaya [64]. All these studies concluded that *Pichia kudriavzevii* and *Wickerhamomyces anomalus* were the most effective yeast preventing *Colletotrichum* infections on fruits when they were applied on the fruit 2–3 h before inoculation with *Colletotrichum* isolates. More recently, Pesce et al. [65] evaluated the antagonistic effect of 241 yeasts recovered from diverse olive and vitivinicultural microenvironments against *C. gloeosporioides* of olive by in vitro and in vivo assays. Among the 241 yeasts tested, nine yeast strains identified as *Candida tropicalis*, *Cryptococcus albidus*, *P. kudriavzevii* and *W. anomalus* were able to control anthracnose when mature olive fruits were artificially inoculated [65].

All these previous results suggest that designing biologic control strategies using bacterial and yeast endophytic strains to prevent *Colletotrichum* infections in almond could be a sustainable alternative for the control of the disease. Studies evaluating the ability of BCAs to enhance natural defenses of the plants should be explored in the coming future.

### 6.4. Chemical Control

The preselection of effective active ingredients under laboratory-controlled conditions (i.e., in vitro sensitivity tests, bioassays on inoculated detached almond fruit or apple) is essential before evaluating fungicides under natural field conditions.

In Israel, Shabi and Katan [19] evaluated 23 fungicides on inoculated detached almond fruit, before and after inoculation with a drop of conidial suspension of *C. acutatum s.l.* (previously reported as *C. gloeosporioides*). The results obtained in this study suggested that curative treatments are not effective since none of the 23 tested fungicides inhibited fruit infection when applied 24 h after inoculation. On the other hand, only four fungicides [captafol, captan and folpet (phthalimide, FRAC group M4) and thiram (EBDC–carbamate; FRAC group M3)] were effective when they were applied 6 h before inoculation [19]. These same authors indicated that three to five protective sprays with captan at 7- to 10-day intervals starting at the petal-fall/fruit-set stage were significantly effective in reducing disease spread.

Adaskaveg et al. [67] also evaluated the effect of nine fungicides against *C. acutatum* under laboratory-controlled conditions in California, with propiconazole and tebuconazole (DMI-triazole; FRAC group 3) being the most effective active ingredients. Subsequently, studies on the chemical control of AA have been conducted in California during the last 20 years in order to determine the most effective fungicides and treatment timing under natural field conditions [22,40,49,68]. All these studies consider fungicide treatment as the most important control strategy to prevent infections, but it should be coupled with cultural practices to achieve the best control. The following fungicide classes were described as the most effective against AA: chloronitrile [FRAC group M5 (i.e., chlorothalonil)]; demethylation (sterol) inhibitors [DMI-triazole; FRAC group 3 (i.e., fenbuconazole, propiconazole, tebuconazole, etc.)]; dicarboximide [FRAC group 2 (i.e., iprodione)], ethylene bisdithiocarbamate [EBDC–carbamate; FRAC group M3 (i.e., mancozeb)], methyl benzimidazole carbamate [MBC; FRAC group 1 (thiophanate-methyl)], phthalimide [FRAC group M4 (captan)], quinone outside inhibitor [QoI–strobilurin; FRAC group 11 (i.e., azoxystrobin, pyraclostrobin, trifloxystrobin, etc.)]; and succinate dehydrogenase inhibitor [SHDI; FRAC group 7 (i.e., boscalid, fluopyram, etc.)].

Concerning the treatment timing, the studies conducted in California suggest that in orchards where anthracnose was damaging in previous years and temperatures are moderate during bloom (18 °C or higher), fungicide sprays must begin at 5%–10% bloom or pink bud and be repeated every 10 to 14 days if rains persist. Otherwise, treatment can begin at petal fall. In all cases, application should be repeated at 7- to 10-day intervals when rains occur during periods of moderate temperatures [22]. These authors recommend to alternate the active ingredients described above making the first application at pink bud using either a DMI or QoI fungicide; the second application at full bloom using premixture (FRAC groups 3/11 or 7/11) fungicide application or mixtures of captan or mancozeb mixed with iprodione or thiophanate–methyl; subsequently, alternate applications of DMI, QoI, captan, chlorothalonil or mancozeb must be conducted as long as conditions are favorable for disease development [22].

More recently, a screening of a broad spectrum of fungicides has been conducted in Spain under laboratory-controlled conditions by in vitro sensitivity tests on PDA amended with the fungicides and bioassays on wounded detached almond fruit and apple fruits inoculated by conidial suspensions of *C. acutatum* and *C. godetiae* [69,70]. All the experiments were conducted with representative isolates of the two *Colletotrichum* species sourced in southern Spain from almonds with symptoms of AA, *C. godetiae* (gray subpopulation) and *C. acutatum* (pink–orange subpopulation) [9]. The results of these laboratory assays were in concordance with the previous studies described above [22] since compounds belonging to the DMI (difenoconazole and tebuconazole), MBC (thiophanate–methyl), phthalimide (folpet) and QoI (azoxystrobin, pyraclostrobin and trifloxystrobin) groups are the most effective [69].

Finally, the most effective fungicides from the laboratory bioassays and several copper-based compounds were evaluated under field conditions in naturally infected fields across the Andalusia region (southern Spain). Field observations suggested that sprays with the protectant folpet or with a commercial premixture of boscalid (FRAC group 7) and pyraclostrobin (FRAC group 11) starting at pink bud resulted in the most effective treatments. Moreover, applications of copper-based compounds in autumn just after leaf falling significantly reduced the DS of AA in the next season [71].

## 7. Conclusions and Future Perspectives

Almond anthracnose caused by *Colletotrichum* spp. is considered a major disease of this crop, and it is endemic in most almond growing regions worldwide. Additionally, it has been considered a re-emerging disease across the Mediterranean basin as a consequence of the new establishment of almond plantings in non-traditional almond-growing areas with climatic, edaphic and orographic conditions favoring crop growing and yield.

The pathogen affects mainly almond fruit showing depressed, round and orange or brown lesions from 5 to 12 mm in diameter that develop on the fruit surface in spring–summer and produce abundant gum. Subsequently, fruit mummify and fall prematurely to the soil.

All the *Colletotrichum* isolates associated with AA over the world has been identified as phylogenetic species within *Colletotrichum acutatum* species complex [*C. acutatum sensu lato* (*s.l.*)] including those from Australia, California, Israel and Spain. Among them, *Colletotrichum fioriniae* and *C. godetiae* are considered the prevalent species causing AA in the main almond-growing regions of the world such as California and Australia. *C. godetiae* has also been described as the prevalent species in southern Spain. Additionally, *C. acutatum s.s*. has been reported as a causal agent of AA in South Australia and Spain.

Severe disease outbreaks are sporadic, occurring only when the optimal conditions for disease development are present. In years of epidemics, the disease may cause important economic losses due to the premature loss of fruit, which could represent up to 80% of the harvest. Likewise, relevant research on the etiology and epidemiology of the disease has been conducted during the past few decades around the world, elucidating the disease cycle of AA and the effects of biotic and abiotic factors on almond infection by *Colletotrichum*.

Significant progress has been made in disease management, especially with regard to fungicides and timing treatments as well as cultivar resistance.

Creating further knowledge on the epidemiology of the disease may be useful to improve the current plant breeding programs to obtain new resistant almond cultivars against AA as well as to modeling the disease. Their application may be useful to schedule and select properly fungicides and timing treatments.

## Figures and Tables

**Table 1:** Grouping of commercial almond cultivars on the basis of their susceptibility to anthracnose caused by *Colletotrichum* spp.*.

Category of Susceptibility	Almond Cultivar
Tolerant	Drake [17], Ferraduel [10], Nonpareil [10,43,44]
Moderately susceptible	Aldrich [17], Carmel [22,44], Constantí [10], Guara [10], Harvey [22], Lauranne [10], Mission [22], NePlus Ultra [22,44], Padre [22], Price [43], Tuono [10], Wood Colony [44]
Susceptible	Antoñeta [10], Belona [10], Butte [22], Diamar [10], Desmayo Largueta [10], Ferragnès [10], Fritz [22], Marcona [10], Marinada [10], Merced [22], Monterey [22], Peerless [17,22], Prays [22], Sonora [17], Soleta [10], Texas [10], Thompson [22], Winters [22]
Highly susceptible	Garrigues [10], Penta [10], Tarraco [10], Vairo [10]

* This table was constructed considering the recent studies on cultivar resistance to AA in the most important almond producing countries of the world: California [17,22,44], Australia [43] and Spain [10].
